# Supplementary material for: Association between cannabis use and result of COVID-19 testing among scholarised adolescents in Andalusia: A cross-sectional study
Source: PLoS One. 2026 May 7;21(5):e0347098. doi: 10.1371/journal.pone.0347098 (PMC13152148; doi:10.1371/journal.pone.0347098)
Supplement: Attachment — Submitted filename: Answer to reviewer 2.docx [file pone.0347098.s002.docx]

REVIEWER 2

Dear Reviewer,

We would like to extend our sincere gratitude for the opportunity to have our article reviewed by experts in your esteemed category. Your suggestions have significantly enhanced the quality of the manuscript, contributing positively to the advancement of science as a whole. The changes have been incorporated into the manuscript (highlighted in yellow - The blue highlighting corresponds to the changes requested by Reviewer 1) and are also detailed in this review document.

Thank you for your valuable contributions.

–

General comments

The manuscript explores a relevant and timely topic: the relationship between COVID-19 and cannabis use among adolescents in Andalusia, Spain. The study aims to understand the factors associated with COVID-19 diagnosis and its connection to cannabis consumption. However, the research, employing a cross-sectional study design with a reasonably large sample size, fails to be methodologically sound. While the article engages in a detailed discussion of the results, I am concerned about their significance given the relatively small sample size of 89 participants, which inherently limits the statistical power of the findings. Furthermore, the contextualisation of the results could be enhanced by incorporating foundational sources, such as the Ministry of Health’s epidemiological documents.

We appreciate your comments. However, the sample is not made up of 89 adolescents. A total of 1,051 adolescents were surveyed, of whom 382 reported having used cannabis at some point in their lives, and 89 reported having been diagnosed with COVID-19.

We understand your concern regarding the small size of positive COVID-19 diagnosis. However, it is important to consider that at that time, healthcare systems were overwhelmed, which may have resulted in many adolescents not receiving a test to diagnose whether they had contracted COVID-19. Additionally, as mentioned in the introduction, diagnosis in the adolescent population is more challenging because they are sometimes asymptomatic. Thus, we believe it is expected for the sample size to be relatively small.

We have tried to improve the contextualization of the results by incorporating foundational sources, such as the Ministry of Health’s epidemiological documents (ESTUDES & EDADES), as you suggested. Thank you.

The study focuses on a specific population of adolescents in four large cities of Western Andalusia, Spain. This is not as clearly stated as one would expect (in particular the urban character of the sample).

It is reflected as follows:

*A random sample was collected from the clusters of 21 high schools from both rural and urban settings of these four provinces.*

Furthermore, this may limit the generalisability of the findings to other regions or populations. At the same time, the specific relevance of these results for Andalusia and beyond are hard to grasp, given the lack of contextualisation on cannabis use among the youth in that region and its specificities (which is however well explained in the ignored EDADES ad ESTUDES studies…)

We frequently use and reference the ESTUDES and EDADES studies in all our research. Therefore, to avoid redundancy, we believe that in this case, citing our previous studies that include this data should suffice. However, we have incorporated the most recent data from both studies related to our study region. These updates have been reflected in the manuscript, where you can also verify the changes highlighted in yellow:

*Among Spanish adolescents aged 14 to 17 years, the prevalence of cannabis use is 20.7%, and increasing to 35.6% at age 18. According to ESTUDES, the average age of initiation of cannabis use in Andalusia is 15.1 years, while EDADES reports it as 18.1 years. The prevalence of cannabis use in Andalusia is 23.3% for lifetime use and 13.6% for use in the last 30 days. Although the ESTUDES study (population aged 14 to 18) shows a declining trend in cannabis use over time, the EDADES study (population aged 15 to 64) indicates an increasing trend from the age of 24 onwards.*

*In 2024, EDADES reports a lifetime prevalence of cannabis use in Andalusia of 27.1%. It is worth noting that in previous reports from 2022, which analyzed the impact of the pandemic, both ESTUDES and EDADES reported a decline in cannabis use, attributing this primarily to the pandemic (e.g., due to mobility restrictions). These data coincide with the OEDA-COVID 2020 survey, which was conducted on the general population residing in Spain, with fieldwork carried out between November 10 and December 3, 2020. This telephone survey identified a change in cannabis consumption patterns, revealing that 3.5% of the population either stopped using cannabis or reduced their consumption during the pandemic, with this percentage being higher among men than women. However, ESTUDES 2022 reported that cannabis use prevalence had progressively increased as social outings, particularly nighttime activities, had resumed.*

*References:*

*National Plan on Drugs of Spain. Survey on drug use in secondary education in Spain (ESTUDES 2022/2023). 2023. Ministry of Health.*

*Spanish Observatory on Drugs and Addictions. 2024 Report (EDADES 2023/2024). Alcohol, Tobacco, and Illegal Drugs in Spain.*

*Spanish Observatory on Drugs and Addictions. OEDA-COVID Survey: Impact of the COVID-19 Pandemic in 2020 on the Pattern of Psychoactive Substance Use and Other Behaviors with Addictive Potential. Madrid: Ministry of Health. Government Delegation for the National Plan on Drugs; 2021. 42 p.*

*National Plan on Drugs of Spain. Survey on drug use in secondary education in Spain (ESTUDES 2021/2022). 2022. Ministry of Health.*

Furthermore, the manuscript relies nn a cross-sectional design, which limits the ability to establish causal relationships between cannabis use and COVID-19. The questions relating to COVID-19 have not been previously validated, which might affect the reliability and validity of the findings. The study found a low Cronbach's alpha of 0.459 for the Rosenberg self-esteem scale, raising concerns about the reliability of the measurement.

We understand your concern regarding the reliability of our measures. Given the novelty of the problem, we did not have the opportunity of validating the COVID-19 questions, what we acknowledge as a limitation of our study. Regarding the Rosenberg self-esteem scales, since it is a previously validated scale and we have followed the guidelines proposed by the author, we found it valuable for the scientific community to be informed about the Cronbach's alpha obtained in our study population. We have already mentioned it as a weakness in the limitation section.

The study may have some implications for public health, especially in the context of the ongoing pandemic and the prevalence of cannabis use among adolescents. However, the recommendations provided by the authors are outdated, lack acknowledgement of the literature and harm reduction approaches, and therefore will have limited relevance for healthcare professionals, educators, and policymakers. The authors should undertake a diligent review of recent literature and address the limitations of the study in more detail, as well as consider addressing the specific type of belief that they describe in more detail.

Thank you for your comment. We have reviewed the recommendations to update them also by reviewing the relevant and recent literature on the topic. Please, see the changes made on the discussion session.

Additional general comments

The manuscript acknowledges the limitations of its cross-sectional design, which makes it impossible to determine causal relationships. However, this could be strengthened, perhaps by mentioning limitations in specific parts of the discussion that refer to the causal nature of an association.

We have reviewed the discussion section based on your comment. Thank you.

The authors use Cronbach’s alpha value for the self-esteem scale that is relatively low (0.459). This should be carefully interpreted and addressed, and perhaps more information should be provided about what this score means.

We have provided information in the discussion about how the low score of the Rosenberg Self-Esteem scale can affect the study. However, it is mercy to say that Rosenberg Scale is one of the most widely used scales for the overall measurement of self-esteem in adolescents and that, despite its widespread acceptance, Cronbach's alpha has significant limitations. For instance, it is sensitive to outliers and the total number of items in a scale.

It has been reflected as follows in the manuscript:

*No studies have been found reporting Cronbach’s alpha as low as 0.5 when applied to adolescents. Such a low reliability coefficient could indicate issues related to scale administration, item comprehension, or cultural adequacy of the instrument. It is possible that the low Cronbach’s alpha is influenced by adolescents’ self-perception of their own self-esteem. Given the sensitive and personal nature of self-esteem, adolescents may struggle to respond honestly, particularly in contexts where expressing vulnerability is socially discouraged. Furthermore, difficulties in instrument administration, such as lack of privacy or social pressure during responses, may also contribute to lower reliability estimates.*

The manuscript could explore the specific types of beliefs held by adolescents (that are described as "not having the belief about cannabis worsening COVID-19") and explore them with more detail.

This recommendation is very interesting. However, given that the study was finalized and only the variables described were included, it is not possible. However, it could be of interest to include this topic on future studies. We added this to the discussion as well as possible belief that could be explored under this topic.

It is reflected as follows:

*Misconceptions about cannabis and COVID-19 may arise from incomplete information, such as media reports suggesting a potential protective effect or the known anti-inflammatory properties of cannabidiol (CBD). Some adolescents might wrongly assume that cannabis consumption enhances immunity or mitigates severe inflammatory responses associated with COVID-19. These misinterpretations highlight the need for clear, evidence-based education on cannabis and its actual health effects.*

*Reference:* *Arboleda, M. (s.f.). Cannabis medicinal y COVID. Recuperado de* [*https://drmaferarboleda.com/cannabis-medicinal-y-covid/*](https://drmaferarboleda.com/cannabis-medicinal-y-covid/)

—

Title page

Title: “addressing its related factors”

Not accurate and not clear.

We have tried to come up with a new title that may be clearer for the reader:

*Exploring Relationships Between Reporting Testing Positive for COVID-19 and Cannabis Use: an Exploratory Study*

–

Abstract

Consistently use “COVID-19” or “covid” but do not alternate arbitrarily between these.

“This study in Andalusia (Spain) surveyed adolescents (14-18 years) and analyzed sociodemographic, psychological, and cannabis use variables, including COVID-19 diagnosis, isolation and some beliefs about cannabis and covid.”

Suggested edits ⇒ “This study in Andalusia (Spain) surveyed [add: “89”] adolescents (14-18 years) and analyzed sociodemographic, psychological, and cannabis use variables, [as well as] COVID-19 diagnosis, isolation and beliefs about cannabis and [COVID-19].”

Thank you. The changes have been made to ensure consistent writing and are reflected in the manuscript. However, in your proposal regarding the sample size, we have added 1,051 adolescents, who were the ones surveyed. Of these, 89 reported having been diagnosed with COVID-19. We also added that only 89 adolescents from the sample were diagnosed as COVID-positive.

Introduction

Page 3 § 3

“Some of the consequences related to cannabis use that may affect COVID-19 may include respiratory issues and social repercussions such as school absenteeism, and family conflicts due to isolations among other reasons”

How does “social repercussions such as school absenteeism, and family conflicts due to isolations” affect COVID-19? What is the correlation between school absenteeism and COViD? At first thought school absenteeism may suggest reduced risks of exposition to contaminations. Not sure if this is the intended meaning. The link betweren “family conflicts” and COVID also seem quite far-fetched. Please either justify what these links are supposed to be, or delete their mention.

Ref 11 is about pulmonary issues. Ref 12 is about social consequences unrelated to COVID. Ref 10 is indeed discussing links between cannabis use and the COVID pandemic but does not seem to justify that there are any link between the prevalence, severity of symptoms, or rate of recovery of COVID-19 and the social consequences of cannabis use mentioned.

We have removed references 10 and 12, and we have replaced it with this sentence and reference is necessary. It is reflected as follows:

*Cannabis use may make it easier to get COVID-19.*

*References:*

*Griffith, N. B., Baker, T. B., Heiden, B. T., Smock, N., Pham, G., Chen, J., ... & Chen, L. S. Cannabis, Tobacco Use, and COVID-19 Outcomes. JAMA Network Open 7.6 (2024): e2417977-e2417977.*

–

Reference 22 is “National Plan on Drugs of Spain. Survey on drug use in secondary education in Spain” dated 2018.

There is a 2023 version of this Ministerial study, undertaken after the COVID.

We believe it is important to keep reference 22 as it is, from 2018/2019, since it was initially used for the design of the questionnaire, even though we have updated the ESTUDES data to the most recent version (2023) in the introduction.

The manuscript uses an outdated 2018 Spanish survey on drug use (ESTUDES). A 2023 version of this study, conducted after the pandemic, is available and should have been included. Additionally, the key Ministry of Health study on drug use, EDADES 2024, is not mentioned. The omission of the 2023 ESTUDES report and the 2024 EDADES study constitutes a significant oversight and a lack of due diligence in the research process. (ESTUDES 2023 https://pnsd.sanidad.gob.es/en/profesionales/sistemasInformacion/sistemaInformacion/pdf/ESTUDES_2023_Informe.pdf ). The key Ministry of Health study on drug use, EDADES 2024, is not even mentioned in the paper. https://pnsd.sanidad.gob.es/profesionales/sistemasInformacion/sistemaInformacion/pdf/2024_Informe_EDADES.pdf

The recent EDADES survey from the National Plan on Drugs confirms several key trends, among others: the prevalence of occasional use is increasing, but the same is not happening with daily use, that of young people, or risk indicators.

This statement from the survey highlights a key finding that is relevant to the manuscript under review. The survey indicates that while occasional cannabis use is becoming more common, daily use, particularly among young people, is not increasing, nor are risk indicators associated with drug use. This oversight is a significant issue because it means that the manuscript lacks the most up-to-date information available on drug use trends in Spain, potentially leading to inaccurate or incomplete conclusions

The mean age of initiation of cannabis use in Spain is 18.4 years (compared to 18.3 in 1995), with the regional mean in Andalusia being 18.1 years. Nationally, the mean age of initiation is 18.1 years for men and 19.0 years for women. The report also notes: “El consumo de cannabis también está más extendido entre el sector masculino, situándose la diferencia porcentual en los 6,8 puntos respecto al dato de las mujeres, diferencia muy similar a la que se registró en la anterior encuesta.”

We really appreciate all your comments and hope to have reached you in the updated and corrected previous comments. Page 3 of the manuscript.

It is also discussed in the discussion section. It is reflected as follows:

*Regarding the prevalence of cannabis use, our results showed that lifetime cannabis use was higher among females than males, while in ESTUDES 2023 showed that lifetime cannabis use was higher among males [11]. Furthermore, looking at the last month cannabis use, while our results showed similar outcomes to those observed over a lifetime cannabis use (i.e., higher consumption in females), ESTUDES survey indicated that the prevalence of cannabis use was nearly twice as high among males (1.7%) compared to females (1%) [11].*

–

In Spain, there are low-dronabinol (delta-9-THC) cannabis products available to the public, particularly CBD products for smoking, vaping, and for oral administration.

In addition, there is a partial regulation of supply to adults (in Spain, there is a self-regulated environment where non-profit associations “Cannabis Social Clubs” have existed for several decades; see Pardal, M., ed. (2022) The Cannabis Social Club, Routledge https://www.routledge.com/The-Cannabis-Social-Club/Pardal/p/book/9780367519087). This has indirect consequences on youth consumption, and it may have been wise for authors to at least briefly comment on it, be it in the limitations section. In addition, a consideration of the approach of harm reduction, would have strengthened the relevance of the paper to contemporary discussions in the field.

We greatly appreciate your suggested approach and will consider it for future studies, as we agree on the importance of the consumption method, cannabis type, and quantity. We have included in the limitations section the next paragraph:

*Moreover, in Spain, as in other countries, there is a partial regulation of cannabis supplies. For instance, low-dronabinol (delta-9-THC) cannabis products are available, which may contribute to a reduced risk perception of cannabis use among youth. Furthermore, considering these trends, new therapeutic approaches focusing on harm reduction are increasingly being adopted by health practitioners and should be considered. Future research could explore these variables to provide a more comprehensive understanding of the relationship between cannabis use and COVID-19 outcomes, as well as potential strategies to mitigate the impact of substance use on infection rates.*

Developing harm reduction in the context of youth substance use: insights from a multi-site qualitative analysis of young people’s harm minimization strategies harmreductionjournal.biomedcentral.com/articles/10.1186/s12954-017-0180-z

Reducing the harms of cannabis use in youth post-legalization: insights from Ontario youth, parents, and service providers harmreductionjournal.biomedcentral.com/articles/10.1186/s12954-024-01112-9

Canadian Centre on Substance Use and Addiction (2020) Talking Pot with Youth: A Cannabis Communication Guide for Youth Allies. https://www.ccsa.ca/talking-pot-youth-cannabis-communication-guide-youth-allies

Fletcher et al.'s European Union Drugs Agency Report: Chapter 13: Young people, recreational drug use and harm reduction. https://www.euda.europa.eu/system/files/media/publications/documents/555/att_101274_EN_emcdda-harm%20red-mon-ch13-web.pdf

Knowledge and Practice of Harm Reduction Strategies Among People Who Report Frequent Cannabis Use journals.sagepub.com/doi/abs/10.1177/1524839920923999?journalCode=hppa

Changes in Alcohol Use during the COVID-19 Pandemic among Young Adults: The Prospective Effect of Anxiety and Depression https://www.mdpi.com/2077-0383/10/19/4468

It is also regrettable that ongoing efforts in Spain in this regard were not acknowledged, e.g. “PSICOCANN, Reducción del Uso de Cannabis y sus Consecuencias en Jóvenes: un Estudio Psicosocial Longitudinal desde la Teoría de la Conducta Planificada y las Estrategias de Protección”. Available from: https://www.researchgate.net/publication/370954344

We have included some of the suggested citations and appreciate the numerous references provided. We are well aware of the cannabis prevention efforts conducted by our Spanish colleagues. However, the length constraints of scientific articles sometimes force us to make difficult choices regarding the focus of our work. (The recommended references that we have not included is because they do not correspond exactly with our target population and we have tried to make this adaptation. Moreover, we have aimed to align the focus of our manuscript with COVID-19 rather than other harms or consequences associated with cannabis use, which have already been described in previously published articles by this research team.)

*References:*

*Parrado-González, A., Vélez-Pérez, L., & Fernández-Calderón, F. Psicocann Project: Technical report of preliminary results. 2023. Ministry of Science and Innovation.*

—

On reference 17.

The “larger study” that this manuscript is based on presents a number of issues, in part the same as mentioned above (using old data and cherry-picking on epidemiological data) in addition to recommending elements such as “focusing on outlining the disadvantages of cannabis use” as a central element in cannabis use disorders prevention among the youth. Yet, decades of research suggest that moralistic, abstinence-oriented messaging may be less efficient in assisting the youth towards cannabis use cessation than other approaches.

“This study is part of a larger investigation into the motivations for cannabis consumption in Andalusian adolescents [17].”

Upon consulting reference 17, I was surprised to encounter the following excerpt:

“Cannabis use in adolescents has increased at a global level (World Drug Report, 2021). In Europe, each country has its own cannabis regulation rules, however its use is only legal in the Netherlands. Spain, and specifically Andalusia due to its proximity to Morocco, has been identified as the main entrance and transit country for cannabis in Europe (European Monitoring Centre for Drugs and Drug Addiction, 2021; Spanish Observatory of Drugs and Addictions, 2021), where cannabis is positioned as the most accessible illegal substance (Spanish National Plan on Drugs, 2018). These data endanger the biopsychosocial health of Andalusian adolescents due to the ease of access to cannabis in the region where they live. Furthermore, 25.5% of Spanish deaths in 2019 were associated with cannabis use. Figures from the Spanish Observatory on Drugs and Addictions (2021) have demonstrated an upward trend of individuals who begin to consume cannabis.”

Firstly, I must point out a factual inaccuracy: cannabis use is not legal in the Netherlands, where its availability is regulated under a tolerated semi-legal framework. Contrastingly, cannabis use is fully legal in Malta. The authors seem to conflate prohibition, decriminalisation, semi-regulated “tolerated” outlets, and fully legally regulated markets. This distinction is critically important, as the regulatory environment significantly influences not only the prevalence of use but also health outcomes associated with drug consumption.

It is a pleasure to engage with your reflections and to reconsider these aspects ourselves as well. Due to the word limitations typically imposed on scientific articles, we aimed to avoid delving too deeply into the terms you mentioned—prohibition, decriminalization, semi-regulated “tolerated” outlets, and fully legally regulated markets. Developing a comprehensive "theory" on cannabis legislation would, in itself, warrant a separate article.

Additionally, we would like to note that part of our research team was Dutch. Nevertheless, we regret if the article previously cited as reference 17 may have caused any confusion. The correct term would have been "decriminalized for personal use". We will contact to the editor for making these changes. We will specify that it is considered a soft drug and, therefore, tolerated for certain recreational uses such as the selling of soft drugs in coffee shops (which is authorized), in contrast to other countries.

<https://www.government.nl/topics/drugs/toleration-policy-regarding-soft-drugs-and-coffee-shops>

Secondly, and more alarmingly, the paragraph includes the statement: “Furthermore, 25.5% of Spanish deaths in 2019 were associated with cannabis use.” This claim is made without any citation or supporting evidence. The notion that over a quarter of fatalities in Spain are attributable to cannabis use is extraordinary and, frankly, implausible. Such an assertion should not only be substantiated but also carefully contextualised if included in an academic work. The lack of clarity surrounding the origin of this statistic – which I suspect may be related to road traffic fatalities involving multiple substances – coupled with the absence of references, renders its inclusion deeply problematic.

It is deeply concerning that this claim was published without proper verification, specificity, or methodological care in handling fatality data – standards that should be expected of academic researchers. The shortcomings in the referenced study’s introduction raise significant questions about its reliability. Given that the manuscript under review is grounded in findings from this “larger study,” its reliance on potentially flawed data and analyses presents a troubling foundation for the current research. In light of these issues, I intend to contact the editors of the cited publication to request a formal correction or retraction of the problematic statements.

We regret that there is no citation in the cited manuscript for this important statement. We provide it here so that you may consult it yourself: Spanish Observatory on Drugs and Addictions. Monograph Cannabis 2022. Consumption and consequences. Madrid: Ministry of Health. Government Delegation for the National Plan on Drugs, 2022.

Link access: chrome-extension://efaidnbmnnnibpcajpcglclefindmkaj/https://pnsd.sanidad.gob.es/profesionales/publicaciones/catalogo/catalogoPNSD/publicaciones/pdf/2022_OEDA_Monografia_Cannabis.pdf

It should have been added to the statement that these deaths were due to acute reactions following cannabis consumption, which is often detected alongside other substances such as sedatives, opioids, cocaine, and/or alcohol.

We will contact to the journal editor to add the remaining citation and clarify the meaning of the suggested phrases to prevent any potential misunderstandings among readers.

Nevertheless,in the current manuscript, we have decided to remove reference 17, as we believe this can help avoid confusion for the reader while we go through the editing process with the other journal, and furthermore, the longitudinal study we referred to in that sentence has been accepted but not yet published.

—

P 4. “The study”

The phrasing of the hypothesis in the sentence, “The hypothesis posits that the diagnosis of COVID-19 among adolescents will associate a positive association with cannabis use,” requires clarification, as its current form is ambiguous and lacks precision. Specifically, the term “associate a positive association” is unclear. Does this refer to a positive epidemiological correlation, participants' positive perceptions (and if so, perceptions of what?), or positive health outcomes? The intended meaning of “association” needs to be explicitly defined to avoid misinterpretation and to ensure that the hypothesis is presented with the necessary specificity and scientific rigour.

Thank you for the opportunity to clear the hypothesis. It is reflected as follows:

*The hypothesis posits that the diagnosis of COVID-19 among adolescents will have a positive epidemiological correlation with cannabis use.*

Furthermore, the objectives are also not the clearest they could be. The stated objectives would benefit from refinement to ensure clarity and precision. For the first objective, “to understand the factors associated with COVID-19 diagnosis in a sample of Andalusian students aged 14 to 18,” did you intend to say something along the lines of “to identify putative cannabis use-related factors associated with COVID-19 diagnoses in a sample of Andalusian students aged 14 to 18”? This phrasing would better align with the context of the study. Similarly, for the second objective, “determining the association between COVID-19 and cannabis consumption through exploring some beliefs related with both terms,” a clearer expression might be “to explore and compare beliefs related to COVID-19 and cannabis use, examining perceived correlations between them.” These adjustments would enhance the precision and readability of the objectives.

We truly appreciate your rephrasing adjustments, which improve the precision and readability of the objectives. Page 4.

–

P. 4.

“67 participants reported sharing cannabis”

Sharing a bag of cannabis and consuming it separately, or sharing a cannabis-containing cigarette, is very different. This sentence does not specify and bundles together cannabis use, cannabis sharing… (plus, in table 3, the OR for sharing cannabis during the pandemic is extremely high. This raises questions about the validity of the measure, and the potential for response bias. Adolescents might underreport sharing cannabis during the pandemic, leading to skewed results. It's also plausible this measure is a proxy for group dynamics that also increase the risk of COVID-19, for example.)

We have deemed it appropriate to include this clarification in the limitations section of the manuscript (page 14).

*A last limitation is the variable "sharing cannabis during the pandemic" should be interpreted with caution, as the self-administered questionnaire did not specify whether it referred to just a single puff, an entire joint, or other details such as a cannabis-containing cigarette. Future studies aiming to investigate the relationship between COVID-19 and shared cannabis use, in order to determine whether it increases transmission, should take these considerations into account.*

–

“A positive COVID-19 diagnosis was associated with higher academic performance, a higher number of isolations due to close contact with positive individuals, and the positive belief that cannabis use would worsen COVID-19.”

I understand that all this is analysing results from 89 paricipans. Statistical relevance questionable.

You are correct, but first, a researcher does not know what they will obtain when conducting research. Secondly, results with lower statistical power also seem important to us, as they help to either improve or, conversely, discontinue exploring a possible association with that particular approach. In our view, for the scientific community, it is better to have data than to lack it.

–

Materials and Methods

It is a significant limitation that the study design and survey instrument did not collect data on the specific types of cannabis products used, whether these were used in conjunction with tobacco, or the methods of cannabis supply. These omissions are regrettable, as these factors could substantially influence the observed outcomes. Variations in product type, joint use with tobacco, and supply methods, may have a considerable impact on the results and their interpretation. This manuscript needs to acknowledge this eventually (perhaps in the limitations section) and certainly may indicate that future research could address these variables to provide a more comprehensive understanding of the relationship between cannabis use and outcomes of COVID-19 symptomatology in adolescents.

The study may suffer from selection bias because it only recruited adolescents from participating schools. Adolescents who do not attend school, or those from schools that did not agree to participate, may have different characteristics. Participants may also underreport cannabis use, especially if they feel stigmatised or are concerned about confidentiality. Finally, participants may have difficulty recalling their exact experiences of cannabis use and COVID-19 diagnosis, which could influence the accuracy of the results.

We have added this paragraph to limitation section (page 14):

*It has been to be considered that the results of this study should be interpreted with caution, as it is based on a self-reported survey (i.e., reporting bias), where data on the specific types of cannabis products (such as herb, resin, CBD) and methods of consumption (such as vaping or oral intake) were not specified.*

—

On Table 1

Table 1 requires substantial revision to enhance its clarity and coherence. The terminology should be reviewed carefully to ensure consistency and accuracy (e.g., replacing "Pandemia" with "pandemic"). Additionally, the headings lack sufficient clarity, and the table conflates declarative beliefs with statistical, demographic, and other types of data. To improve the structure, it is essential to distinguish between objective data (e.g., statistical and demographic information) and subjective data (e.g., beliefs and perceptions). Clear categorisation and separation of these elements would significantly improve the table’s readability and utility for the reader.

We have replacing “pandemia” for “pandemic”. We are sorry for the mistake, and we also included subtitles on table 1 so the reader could distinguish easily the objective data from the subjective one. Furthermore, we have also mentioned in the text. It is reflected as follows on page 8:

*For better comprehension, the variables were divided into two groups. On one hand, the objective variables, which include sociodemographic data such as age or the number of isolations. On the other hand, the more subjective variables related to COVID-19, referring to the adolescent's beliefs and perceptions.*

—

Results

A relatively small percentage (8.5%) of the participants had a positive COVID-19 diagnosis. The study’s findings, especially the association with higher academic performance, require careful interpretation.

A significant proportion of the sample (36.4%) had used cannabis at some point in their lives, while 15.6% had used it in the last year, and 14.8% in the last month. The authors find that the personality trait of sensation seeking was significantly associated with cannabis use across all three periods, with impulsivity also associated with use in the last year and month. Age was positively associated with lifetime cannabis use. However, a discussion on the methods and types of cannabis consumption (including the product used, whether herb, resin, information about adulteration, about dronabinol and/or CBD contents, and critically: mixture or not with tobacco) is missing.

COVID-19 and Cannabis Use: A positive association was found between COVID-19 diagnosis and cannabis use in the last year and month. Users were less likely to believe that cannabis smoke spreads COVID-19 or that cannabis use worsens COVID-19. The number of isolations was also higher among users in the last year and the last month.

Logistic Regression: The regression analysis showed that sharing cannabis during the pandemic and not believing that cannabis worsens COVID-19 were related to cannabis use across all measured time frames. A diagnosis of COVID-19 was also associated with cannabis use in the last month. The Nagelkerke's R-squared value suggests that the model explains a decent amount of variance for last month cannabis use (40%).

—

On Table 3

The results in Table 3 are derived from a cross-sectional study. While Table 3 presents interesting findings, these should be interpreted cautiously due to the limitations of the cross-sectional study design and potential biases.

While the authors use logistic regression to explore associations, they can’t definitively say that one variable causes a change in another. Also, the regression analysis controls for sociodemographic and psychological variables; but there may be other unmeasured confounding factors influencing the results (e.g. the study doesn’t explore the social environment in which cannabis use takes place, which could significantly impact both COVID-19 risk and cannabis use patterns).

We have included the next paragraph in the limitations section (page 14):

*Although the regression analysis controls for soinciodemographic and psychological variables, there may be other unmeasured confounding factors influencing the results (e.g., the social environment in which cannabis use occurs, which could significantly impact both COVID-19 risk, and cannabis use patterns).*

It has been also reflected in a discussion section (page 11):

*Again, these results should be interpreted with caution, as the exceptionally high OR values may suggest a correlation with other unmeasured variables, such as cannabis’ products, where cannabis take place, family dynamics or mental health that may influence the relationship between COVID-19 and cannabis use.*

The odd ratios in Table 3 suggest the likelihood of cannabis use (lifetime, last year, last month) given the presence of various factors. ORs should be interpreted with caution, particularly when they are large: (as mentioned earlier) the OR for “Share Cannabis During Pandemic” is extremely high, which could indicate that this variable is correlated with other unmeasured variables; the OR for the last month COVID-19 diagnosis is 2.898, suggesting that individuals with a diagnosis of COVID-19 are almost 3 times more likely to use cannabis in the last month, which is an association that deserves further scrutiny. But it cannot be stated this is a causal factor due to the research design.

It has been reflected as follows in the discussion section:

*Regarding the estimated association between reporting testing positive in COVID-19 and using cannabis,* *the OR for cannabis use in the last month and COVID-19 testing positive was 2.898, suggesting that individuals diagnosed with COVID-19 are almost three times more likely to have used cannabis in the last month.* *It may be because of a combination of psychological stressors and coping mechanisms: the fear of COVID-19 testing positive has been linked to increased cannabis use, as they often turn to cannabis to manage anxiety [30]. Yet, no studies were found that associated cannabis use with COVID-19 testing positive**.*

New reference:

Diggs BA, Baral A, Hlaing W, Pan Y, Camacho-Rivera M, Ortega J, Vidot DC. Fear of COVID-19 Diagnosis and Cannabis Use Patterns Among Adults with Chronic Health Conditions: Results from COVID-19 Cannabis Health Study. J Psychoactive Drugs. 2024 Oct 28:1-10. doi: 10.1080/02791072.2024.2421903. Epub ahead of print. PMID: 39468475.

In Table 3, some CIs are quite wide (e.g. relationship of COVID-19 and lifetime cannabis use: 1.1425 to 3.1918). Some CIs lack statistical significance, as in the case of last month cannabis use. Likewise, the CIs for the variable “Dx COVID-19” and “lifetime” and “last year” cannabis use are statistically insignificant in this analysis.

On “Cannabis Worsens COVID-19” the OR suggests that those who do not believe that cannabis worsens COVID-19 are more likely to use cannabis. This aligns with the findings but doesn’t necessarily mean that this belief causes cannabis use. It may be that those who use cannabis are more likely to justify or minimise the potential harm, meaning that the direction of this association may be the other way round. This requires careful consideration in the discussion section.

The relationship of the “Dx COVID-19 (yes VS no)” association also needs further interpretation in the discussion section.

It is reflected as follows, in two different paragraphs of the discussion section (page 12)

The Nagelkerke R2 values indicate that 60% of the variability is explained by factors not included in the model; the model is further considerably less effective in explaining variability in lifetime and last year cannabis use.

It is reflected as follows, in the limitation section:

*Additionally, although the regression analysis controls for sociodemographic and psychological variables, there may be other unmeasured confounding factors influencing the results (e.g., the social environment in which cannabis use occurs, which could significantly impact both COVID-19 risk, or other factors such as cannabis use patterns, cannabis’ products, family dynamics or mental health). In fact, the multivariate analysis suggests that 60% of the variability is attributed to factors not accounted for in the model, especially in the case of lifetime and last-year cannabis use. Thus, caution is requested in the interpretation of the data, as well as when interpreting the confidence coefficients (some intervals were wide, reducing the statistical significance).*

Potential biases include: the selection bias (The study may suffer from selection bias because it only recruited adolescents from participating schools. Adolescents who do not attend school, or those from schools that did not agree to participate, may have different characteristics); the reporting bias (where participants may under-report cannabis use, especially if they feel stigmatised or are concerned about confidentiality); or the recall bias (participants might have difficulty recalling their exact experiences of cannabis use and COVID-19 diagnosis, which could influence the accuracy of the results)

It is recommended that authors better acknowledge limitations in more detail, particularly the ones associated with cross-sectional data and the possibility of confounding factors, interpret the data with caution, avoiding causal language when discussing associations and focusing on describing the associations as they are (refraining from claiming a causal direction where it isn’t statistically supported). Authors would also be well advised to explore possible additional variables, or at least indicate in their discussion section that future research should explore additional factors that may influence the relationship between COVID-19 and cannabis use, such as social context, mental health, and family dynamics.

It has been reflected in the discussion section as follows:

*At last, it should be considered that as the adolescents' responses are based on perceptions rather than experiences, it should also be considered that cannabis users may respond in a socially desirable manner, aligning with perceived health norms to minimize potential health harm. Nevertheless, and given the estimated association found, it is important that educators and nurses explain to them that the consequences of having COVID-19 while using cannabis could be detrimental for health.* *It would also be relevant to study the differences between the effects of CBD and THC separately to obtain clearer conclusions, as some existing literature suggests that CBD could be a protective factor against COVID-19 [43].*

It is also reflected in the limitation section:

*A second limitation is that, due to the contemporary nature of the study topic, the questions related to COVID-19 have not been previously validated (potential recall bias or tic errors).*

—

Discussion

Gaps in Research

The authors acknowledge a gap in research regarding beliefs about cannabis and COVID-19, noting a lack of studies examining the beliefs about smoke and transmission and cannabis worsening COVID-19. However, the starting hypothesis that cannabis use worsens COVID effects is not justified or substantiated in any moment. The complex pharmacological interaction between tobacco and cannabis and the differentiated effects of both on the human body, as well as different correlations with COVID-19 symptoms, is totally missing from the paper.

The study's hypothesis that a COVID-19 diagnosis among adolescents will have a positive association with cannabis use is indeed supported by the data. However, the idea that cannabis use worsens COVID symptoms of effects is not clearly established in the study or elsewhere. The authors do not provide any tentative pharmacological explanation for this. It is crucial to approach such hypotheses with caution and rely on comprehensive scientific evidence when considering health-related behaviours. The study does not explore the complex interactions between cannabis and tobacco use, nor the differentiated effects of these substances on the body. There is a lack of discussion about how cannabis and tobacco may affect the lungs or other body systems, and how this may impact COVID-19. This is particularly concerning given the findings that indicate that dronabinol (delta-9-THC), a principal component of cannabis, may induce short-term bronchodilation. Studies have observed increases of 0.15–0.25 litres in forced expiratory volume in one second (FEV₁) following THC administration (https://pmc.ncbi.nlm.nih.gov/articles/PMC2720277/). Conversely, chronic cannabis smoking (but not occasional) may be associated with respiratory symptoms and airway irritations (https://www.nature.com/articles/npjpcrm201671).

Leitzke M. (2023) Is the post-COVID-19 syndrome a severe impairment of acetylcholine-orchestrated neuromodulation that responds to nicotine administration?. Bioelectron Med, 9(1): 2. doi:10.1186/s42234-023-00104-7 <https://bioelecmed.biomedcentral.com/articles/10.1186/s42234-023-00104-7>

Thank you for your comment. We decided not to introduce the discussion about how cannabis and tobacco may affect the lungs of other body system, and how this may impact COVID-19. Our focus is not on discussing the effects of COVID-19, as our study does not include sufficient variables to measure them. Rather, with the data we were able to include in the survey in 2020, we aimed to explore the potential relationship between cannabis use and COVID-19 diagnosis, rather than the effects of the disease itself. Therefore, we do not address symptomatology, pharmacological treatment, or tobacco use. Nonetheless, we have included some reflections that may help future studies pursue the approach you suggest. It is reflected as follows:

*Although our study cannot conclude that cannabis use worsens COVID symptoms, a study (n=13,099) of adults concluded that the frequency of cannabis use could be considered a possible predictor of COVID-19 mortality risk [34].*

*It would also be relevant to study the differences between the effects of CBD and THC separately to obtain clearer conclusions, as some existing literature suggests that CBD could be a protective factor against COVID-19.*

We have also changed some parts of the introduction to make our approach more understandable.

—

Conclusion

The conclusion presented in the manuscript attempts to concisely summarise the main findings. However, the conclusion would benefit from greater clarity, neutrality, and cautious phrasing to strengthen its scientific rigour. As it stands, the language appears overly assertive and lacks the balanced tone required to reflect the study’s findings appropriately. For instance, phrases such as "diagnosing COVID-19 in adolescents who use cannabis is crucial" imply causality and urgency that may not be fully supported by the data. The conclusions should avoid overgeneralisation and instead frame the findings with appropriate & cautiously-chosen qualifiers, while also acknowledging the limitations of the study (such as sample size, or potential confounders). Lastly, the phrasing would benefit from a more neutral and precise presentation to enhance readability and professionalism.

We have removed the word *crucial* throughout the manuscript and replaced it with synonyms that do not imply causality.

–

Additional comments on neutral and non-stigmatising terminology. Below are additional comments I tend to include when reviewing manuscripts which refer to controlled drugs such as cannabis: The manuscript should be carefully reviewed to ensure the use of neutral and non-stigmatizing language. The following references offer guidance on avoiding stigmatising language in academic writing:

- Guiding principles for breaking down drug-related stigma in academic writing (International Journal of Drug Policy, 2024) https://doi.org/10.1016/j.drugpo.2024.104515

- Access to treatment with controlled medicines rationale and recommendations for neutral, precise, and respectful language (Public Health, 2017) http://dx.doi.org/10.1016/j.puhe.2017.08.021

- Confronting Inadvertent Stigma and Pejorative Language in Addiction Scholarship: A Recognition and Response (Substance Abuse, 2018) https://pmc.ncbi.nlm.nih.gov/articles/PMC6042508/

We appreciate the guidance provided on avoiding stigmatizing language in academic writing. Undoubtedly, language is a barrier for those of us who are not native English speakers, and we hope to have corrected such language throughout the manuscript.

For example:

*Diagnosing COVID-19 in adolescents who use cannabis is important, as the use of this substance may impact both respiratory and immune systems, potentially influencing infection severity, recovery trajectory, and the ease of viral transmission.*

—

In conclusion, while the study explores a relevant and timely topic, it fails to meet the necessary standards of scientific rigour for publication in PLOS ONE as such. The manuscript's primary limitations include:

- The use of outdated epidemiological data, specifically from the 2018 Spanish National Plan on Drugs survey (ESTUDES), neglecting more recent and relevant studies such as the 2023 ESTUDES report and the 2024 EDADES report. This failure to incorporate the most current data undermines the study's contextualisation and relevance.

- A lack of precision and clarity in the formulation of the research questions and the interpretation of results, for example, using the term "students" when "adolescents" may be more appropriate, the poorly defined hypothesis, and ambiguous wording concerning cannabis sharing.

- An over-reliance on a small sample of participants who tested positive for COVID-19 (N=89), which limits the statistical significance and generalizability of the findings.

It is reflected in limitation sections as follows:

*Furthermore, the study's reliance on a limited sample of participants who tested positive for COVID-19 (N=89) restricts the statistical power and generalizability of the findings.*

- A problematic mixing of objective and subjective data, most evident in Table 1, which makes interpretation difficult.

- The lack of a thorough discussion of harm reduction strategies, which have been proven to be effective and should be considered when addressing youth cannabis use

- A tendency to draw strong conclusions from weak associations, such as the suggestion of links between school absenteeism and COVID-19.

Overall, the manuscript needs substantial revision to address these critical issues. A more robust methodology, clearer research questions, up-to-date data, and a more nuanced discussion of the findings are required before this study can be considered a valid contribution to the scientific record. Without these fundamental improvements, the conclusions drawn from the data remain questionable.

We have included new sentences to improve the quality of the manuscript. They are highlighted in yellow but not all of them do appear in this document. In addition, the most important information has already been reflected in this document in italic writing style. We advised to read the new changes from the manuscript, especially the discussion section.

We deeply appreciate the time and effort dedicated to reviewing our work, as well as the opportunity to improve its rigor and precision. We have carefully considered each of the comments and have worked to implement the suggested changes wherever possible. However, we would like to point out that some of the terms used in the review seemed to us less appropriate within the framework of constructive scientific critique. We firmly believe that in the scientific community, rigor should always be accompanied by mutual respect, recognizing the effort and dedication of all researchers, regardless of their career stage or experience.

We trust that the modifications made contribute to a stronger version of the manuscript and remain open to any further suggestions that may help improve its quality.

On behalf of all the authors.
