# Supplementary material for: Association between cannabis use and result of COVID-19 testing among scholarised adolescents in Andalusia: A cross-sectional study
Source: PLoS One. 2026 May 7;21(5):e0347098. doi: 10.1371/journal.pone.0347098 (PMC13152148; doi:10.1371/journal.pone.0347098)
Supplement: Attachment — Submitted filename: Answer_round2_Plsone.docx [file pone.0347098.s003.docx]

**REVIEWER 1**

We sincerely thank Reviewer #1 for their thoughtful and constructive feedback on the revised version of our manuscript. We appreciate the positive remarks regarding the improvements made, including the inclusion of the STROBE checklist, detailed age distribution, and clarification of the consent procedures. Below, we address each of the remaining concerns point by point.

**1. Causal Language in the Conclusion and Interpretation of Results**

**Reviewer’s comment:**
The reviewer correctly notes that some statements in the conclusion still imply causality, which is not appropriate for a cross-sectional design. The reviewer suggests using more cautious phrasing such as “An estimated association was observed between…” or “Data showed a correlation between…”.

**Response:**
We fully agree with the reviewer’s observation and have revised the relevant sections of the manuscript to ensure that all interpretations are expressed in non-causal terms. Specifically, the statement in the conclusion now reads:

“*An estimated association was observed between COVID-19 positivity and cannabis use, particularly last-month consumption. Data also showed correlations with higher academic performance, a greater number of isolations, and the perception that cannabis consumption worsens COVID-19 outcomes*.”

We have carefully reviewed the entire manuscript to ensure consistent use of language that accurately reflects the cross-sectional nature of the study and avoids any implication of causality.

**2. Introduction – Misleading Interpretation of the Literature**

**Reviewer’s comment:**
The reviewer points out that our citation of Griffith et al. (2024) could be interpreted as overstating the evidence, as the cited study reports associations with hospitalization and ICU admission rather than directly with infection risk.

**Response:**
We thank the reviewer for this valuable clarification. We have revised the text in the introduction to more accurately reflect the findings of Griffith et al. (2024). The revised sentence now reads:

*“Previous studies, such as that by Griffith et al. (2024), have reported that cannabis use was associated with an increased risk of hospitalization and ICU admission among individuals with COVID-19.”*

We believe this modification provides a more faithful representation of the original study and avoids overstating its conclusions. The corresponding reference has been retained and properly contextualized within our discussion.

**REVIEWER 4**

We sincerely thank Reviewer #4 for their helpful and constructive feedback, which has guided further refinement of our statistical analysis and presentation.

**Comment:**
The reviewer suggests providing more details on the model-building strategies used for Table 3, considering parsimonious models for each cannabis use category, exploring mutually exclusive cannabis-use groups with multinomial logistic regression, and including 95% confidence intervals for the odds ratios.

**Response:**
We appreciate these valuable suggestions:

1. **Model-Building Strategy and Parsimonious Models and Cannabis Use Categories:**
   We appreciate your suggestion; however, this was not the objective of our study. Our aim was not to identify the most parsimonious model, but rather to conduct an exploration analysis by retaining all variables in order to examine whether any associations existed. Nevertheless, we have included in the limitation section that in future studies that consideration should be taking into account. It is reflected as follows:

*“**They also could consider reclassifying participants into mutually exclusive groups (no use = 669; last-month use = 156; use more than one month ago = 226) and conducting supplementary multinomial logistic regression analyses.”*

2. **95% Confidence Intervals:**
   We have revised Table 3 and the 95% confidence intervals has been already included for all reported odds ratios, as suggested. We agree that this addition substantially enhances the interpretability and statistical transparency of the results.

We thank the reviewer for these insightful recommendations, which have strengthened both the methodological rigor and clarity of our results.
