# Supplementary material for: Association between cannabis use and result of COVID-19 testing among scholarised adolescents in Andalusia: A cross-sectional study
Source: PLoS One. 2026 May 7;21(5):e0347098. doi: 10.1371/journal.pone.0347098 (PMC13152148; doi:10.1371/journal.pone.0347098)
Supplement: Attachment — Submitted filename: Response to Reviewers_3rdRound_mls.docx [file pone.0347098.s004.docx]

**Reviewer #1:**

 I would like to thank the authors for the substantial improvements made throughout the revision process. The manuscript is now clearer, more consistent, and the main concerns raised in previous rounds have been adequately addressed. The adjustments to causal language, clarification of the introduction, and revisions throughout the text have strengthened the overall quality of the work.

At this point, I believe the manuscript is suitable for publication pending only minor formatting adjustments. Several tables still present formatting inconsistencies, including:

Table 1 appearing split across two pages;

Placeholder text such as “insertheretable1”, “insertheretable2”, and “ insertheretable3” remaining in the manuscript.

I kindly recommend a final formatting check to ensure that all tables appear as single, complete blocks and that placeholders are removed. These adjustments are minor and do not affect the scientific content of the manuscript.

Response from authors:

We thank reviewer 1 for their comments. We have removed the 'insert Table 1 here' in all of the table’s placeholder, and Table 1 now appears on a single page.

**Reviewer #2:**

I thank the authors for their work and for their involvement in the review process. I also take the opportunity to wish them all an happy new year.

I note the authors have "carefully reviewed the entire manuscript to ensure consistent use of language that accurately reflects the cross-sectional nature of the study and avoids any implication of causality." While this should not have required two rounds of peer-review, it is indeed finally appropriate in the body of the manuscript; it is, however not, in the Abstract. My review focuses therefore on the Abstract, as well as the introduction.

An outstanding element that deserve address is the apparent deterritorialisation of the results, which does not seem justified. This study was conducted in four specific urban contexts of Western Andalucía, and among adolescents. None of these elements are present in the title (geographical location [Andalucia], type of environment [urban], population studied [scholarised adolescents]). It is critical that these elements appear in the title, and clearly articulated in the abstract.

The importance of accurately representing the specificities of the localised regional context within which the study was conducted (urban context in Western Andalucía) is key.
I also stress that the territory in question (four specific large cities in Western Andalucía) is located at the najor entry point of cannabis resin for illicit markets, fromNorthern Africa to Europe. This absolutely unique situation is nowhere mentioned. Other geographical, socio-cultural, and political contextual elements may have been welcome.
Furthermore, I note that imported cannabis drugs from that Northern African region often constitute “hashish” (cannabis resin) and not “flowers” (dry herbal tops) which are the subject of most published research. Besides possible differences in effects on individual and public health, the consumption of hashish is, I believe, directly associated with that of tobacco. I may have missed it, but this critical element was not mentioned in the manuscript. Finally, there may be specific rituals of collective use and sharing associated with “hashish” that are not with “flowers”.
Tobacco consumption, type of product, use rituals, excess quantity on the market due to the major EU zone of transit… PLOS One has an international audience, not expected to be aware of these elements, and which should therefore be mentioned.

We sincerely thank the reviewer for the careful, detailed, and constructive feedback. We appreciate the emphasis on contextual precision, conceptual clarity, and restraint in interpretation.

Unfortunately, we have not included additional details regarding specific product characteristics (such as resin mixed with tobacco, imports, or market trends). This is because such factors were outside the original scope of our research; furthermore, the participants were not specifically surveyed about their exact methods of consumption. In fact, the questions was “How many days have you used marijuana (cannabis, weed, hash, joint)?”, mixing the different types of consumption. Consequently, we feel this data does not align with the current manuscript's objectives. Nevertheless, we sincerely appreciate this insightful observation and have noted it as a consideration for future studies.

We have included the following paragraph in the introduction to provide better context:

Approximately 94% of users consume cannabis in the form of hand-rolled cigarettes. In Spain, these are typically prepared by mixing cannabis (frequently in the form of hashish) with tobacco

TITLE
I suggest deletion of initial "the" in the title, as in "Exploring Relationships Between".
I also respectfully propose to add language along the lines of: "among 89 Adolescents in Andalusian Cities." at the end.
I have the memory of an original title being perhaps more specific in that regard (but I could not locate it on the editorial manager).

Thank you for your comments. We have reviewed the title considering them, the new title is:

*Association Between Cannabis Use and Result of COVID-19 Testing Among Scholarised Adolescents in Andalusia: a Cross-Sectional Study*

ABSTRACT — Methods

"This study conducted in Andalusia, Spain, surveyed 1,051 adolescents aged 14 to 18 years, of whom 89 reported testing positive for COVID-19."

I continue to believe that the mention of the figure 1,051 is not relevant in the abstract —no more than the totla figure of adolescents in Westedn Andalucia. This is an article about positive results from a study of 89 participants. The abstract must reflect this in a non-misleading way. Given that the rest of the article refers to "our sample" without making clear that sample is 89, not 1,051.
The figure 1,051 is superfluous.

We appreciate the concern regarding potential ambiguity. However, in our analysis we compared the adolescents that inform of a positive result with they that did not inform a positive result. So, we think that we need to recognize also the larger sample.

It is reflected as follows:

This study conducted in Andalusia, Spain. Although, 1,051 adolescents aged 14 to 18 years were included in this cross-sectional study, of these, the 89 (8.5%) reported testing positive for COVID-19, who were the main target for our analyses.

ABSTRACT — Results

"Sharing cannabis during the pandemic and the lack of perception that cannabis use exacerbates COVID-19 are related to cannabis use"

Did the authors intended to mean "is related to cannabis use" (or "related to…") instead of "are"? If so, the meaning changes drastically.
If not, the authors are saying with this sentence:
1) Sharing cannabis during the pandemic … is related to cannabis use — a wholly tautological statement.
2) lack of perception that cannabis use exacerbates COVID-19 … is related to cannabis use — a proper finding.

(1) is superfluous. The fact that (1) is merged with the entirely different (2) is concerning.

Thank you for highlighting this issue. The sentence has been rewritten to avoid ambiguity and tautology. We have removed the phrasing that could be interpreted as circular.

The revised sentence now states:

Adolescents testing positive for COVID-19 are almost 2.89 times more likely to have used cannabis in the last month. Additionally, number of isolations was higher among cannabis users and positive testing. Cannabis users showed a lack of perceived risk that cannabis use could exacerbate COVID-19 and that cannabis’ smoke could spread COVID-19. Sharing cannabis among adolescents during the pandemic may increase the risk of COVID-19 transmission.

ABSTRACT — Conclusions

This section also requires considerable revision and restraint. Particularly, the latter sentence is perhaps superfluous or overstated. This paper does not in any way bring "comprehensive" knoelwedge, just additional elements. I must again note a regrettable tendency towards overstatement throughout.

These are signaling a certain lack of ability to communicate results in an accurate and transparent manner. Abstracts are critical parts of the research. I reiterate my call for a comprehensive rewriting of the abstract and title, keeping in mind modesty and the scope of these results: this is a study of 89 cases in 4 large cities in half of one region of Spain. It is an extremely insightful, interesting, well-conducted, and useful study. But it is not what it is not, and must focus on what it is.

We agree that the previous wording overstated the contribution.

The conclusion has been substantially rewritten to reflect:

- The exploratory and cross-sectional nature of the study
- The limited sample size
- The contextual specificity

Any reference to “comprehensive knowledge” has been removed. The conclusions are now framed as providing additional contextualized evidence rather than broad claims.

It is reflected as follows:

Informing whether adolescents who use cannabis may be more likely to test positive for COVID-19 can contribute to a better understanding of substance use patterns during a public health emergency. However, given the exploratory and cross-sectional nature of this study, as well as its limited and context-specific sample, the findings should be interpreted with caution. Despite this, we believe that this research may inform future studies exploring the impact of cannabis use among adolescents during public emergency situations in larger samples and other contexts.

INTRODUCTION §1

This opening is structurally problematic. It starts by talking about the WHO’s opinion on “the pandemic” without having qualified it prior.
In my opinion, this introduction ought to start with the first sentence from the M&M section (i.e., “In March 2020, a lockdown…” up to “… of our adolescents”), followed by the present sentence about WHO and end of pandemic period.
This would be a serious, solid, clear way to begin the article, and would allow to avoid subsequent repetitions (hence reducing the length of the introduction andM&M section).

We agree with the structural suggestion.

The Introduction has been reorganized so that:

- The contextual description of lockdown in March 2020 now appears at the beginning.
- The WHO declaration is introduced after situating the timeline.
- Redundancies between Introduction and Methods have been eliminated.

INTRODUCTION §2
"The global increase in drug seizures in 2020 intensified during the coronavirus disease (COVID-19) pandemic in many countries [5]. In fact, the United Nations' Annual Report for 2020 against drug abuse highlighted that most countries reported an increase in cannabis consumption during the COVID-19 pandemic [6]."
Seizures are only reflective of one thing: police zeal and activity. Stop funding police, seizures go down: does that mean drug use goes down? It is inappropriate to conflate such distinct data in this sentence. Delete the paragraph, for global seizure data serve no analytical purpose. The only use of seizure data may be to address the hashish-contet mentioned elsewhere (but I assume this would not go in §2). Otherwise, this sentence and its discussion of global police activity has no room in a paper about adolescents in four specific urban contexts of Western Andalucía.

Provided that this entire paragraph adds nothing to the topic, and conflates broad data, I do not see any better solution than deleting it.

We accept the reviewer’s concern regarding the interpretative limitations of seizure data.

The paragraph referencing global seizure trends has been removed, as it did not directly serve the analytical aims of the manuscript.

INTRODUCTION §3
"Cannabis consumption poses a threat to public health, since it stands as the most widely used illicit drug among teenagers globally [7]."
Prevalence alone does not constitute evidence of a “public health threat.” UNODC World Drug Report, the source cited, explicitly distinguishes between prevalence of use and of cannabis use disorders. Cannabis use disorders represent a subset of cannabis consumption. Failing to acknowledge this distinction, which is explicit in the cited source, while conflating widespread use with public health harm, is conceptually incorrect and misleading.
I would like to highlight that this seems to constitute a persistent trend in conflating prevalence, harm, simple use and use disorder, and causality — which apparently remain uncorrected despite repeated and explicit reviewer feedback across multiple rounds of review.

From a broader perspective, this paragraph does not serve the stated aims of the manuscript. The article does not examine cannabis use as a public health risk per se, but rather the association between cannabis use and reporting testing positive for COVID-19. As such, general statements about cannabis as a public health threat unrelated to COVID-19 (or possibly other similar infectious and/or lung-related health conditions) are not analytically relevant in this context.

The paragraph would be better aligned with the study objectives if it focused directly on prior evidence linking cannabis use and COVID-19 outcomes, followed by relevant prevalence data to contextualize the population studied (global → European → Spanish adolescents):

(sentence 1 of the new §) Previous studies, such as that by Griffith et al. (2024), have reported that cannabis use was associated with an increased risk of hospitalization and ICU admission among individuals with COVID-19. (sentence 2 of the new §) The global prevalence of cannabis use in the last year was 5.8% (corresponding to 12 million) among 15- and 16-year-old adolescents [8], with the highest prevalence worldwide observed in European youths aged 15 to 24 [6,9] (sentence 3 of the new §) move on with data about Spain’s consumption patterns among the youth, currently in the next paragraph (4th)

We appreciate this important conceptual clarification.

The paragraph has been revised to:

- Avoid conflating prevalence with harm or use disorder.
- Remove generalized statements framing cannabis use as inherently a “public health threat.”
- Focus directly on prior evidence linking cannabis use with COVID-19 outcomes.
- Present prevalence data in a structured progression, as suggested.

INTRODUCTION §4
Suggested merger with revised §3 as proposed above.
This does not apply to the final part of the paragraph: “the EDADES study (population aged 15 to 64) indicates an increasing trend from the age of 24 onwards”. If this article is about adolescents under 24, there are little reasons to mention this datum.

We have removed the reference to trends in populations aged 24+ years, as it was not directly relevant to an adolescent sample.

M&M
This section ought to refer to the specificities of the territory (4 large cities) and the product (resin mixed with tobacco, imports, large market), as mentioned above.

We fully agree that the localized context is essential for an international readership.

In the revised manuscript, we have:

- Explicitly clarified that the study was conducted in four major areas in Western Andalusia. However, the reviewer must consider that the study was done through urban and rural areas in this territory.
- Added contextual information regarding the region’s role as a major entry point for cannabis resin trafficking from Northern Africa into Europe.

CONCLUSION.

"The findings of this exploratory study give light to possible interventions to reduce the harm of cannabis use in a pandemic situation"
Replace "the harm of cannabis" by "harms associated with cannabis use" you may want to add elements of context. This paper offers limited guidance on reducing the harms of pure flowers vaporised in, say, Hawaii. It serves for tobacco-hashcish mix in adolescents in Europe.
I would not recommend accepting a final version which would retain the mention of "interventions" as it currently stands. This article gives absolutely no insight for practical intervention. Stating so is a large leap. A premature one. It would be concerning if real-world public health interventions were based solely on such cross-sectional studies of small samples of urban adolescents.
Consider something along the lines of: "The findings of this exploratory study provide additional data in relation to the harms associated with the use of cannabis resin among adolescents, in this specific context, in a situation of public health emergency like the COVID-19 pandemic, and may provide insight in the design of public health interventions."

I am conscious of the discomfort this long review process may have caused to the authors, but I am confident that integrating these improvements will make for a paper reduced in its scope, but dramatically increased in its quality — which will benefit both public health and the authors' track record.

I thank you and am confident the authors' revised version will be good for publication.

We agree that the previous formulation was overstated.

Changes made:

- Replaced “the harm of cannabis” with “harms associated with cannabis use.”
- Removed direct claims about informing interventions.
- Reframed the final statement more cautiously.

The revised conclusion now reads:

The findings of this exploratory study provide additional context-specific data regarding cannabis use among adolescents in Western Andalusia during the COVID-19 pandemic. An estimated association was observed between COVID-19 positivity and cannabis use, particularly last-month consumption where the odds was almost three points higher. Moreover, cannabis users showed a slightly lower perception that cannabis’ smoke could spread COVID-19 while non-cannabis users reported having favorable perceptions about cannabis use could worsen COVID-19. This perception is also higher in those that were positive testing COVID-19. Finally, the mean number of isolations was higher among cannabis users and positive testing. As consuming cannabis has been identified as a predictor of COVID-19 hospitalization and mortality risk we think that our study gives some context-insights that could be relevant for preparing for future public health emergencies and for informed future large scale research. It is also important of leverage the conscientious about the effect of cannabis use among adolescents and their context, which can be exacerbated in a pandemic situation.

We believe this formulation better reflects the modest scope of the study.

We are grateful for the careful review and believe the manuscript is significantly improved as a result. We thank the reviewer again for the thorough and constructive evaluation, which has strengthened the manuscript both conceptually and structurally.

**Reviewer #3:**

I am happy having the opportunity to read and review the manuscript assigned to me, titled “Exploring the Relationships Between Reporting Testing Positive for COVID-19 and Cannabis Use: an Exploratory Study” submitted to Plos One.
I have carefully reviewed this manuscript and below is my decision.
The paper is well-suited for publication in the Plos One. All comments are well-improved the quality of paper. Good luck!

Response from authors:

We sincerely thank the reviewer for their positive feedback and for the time dedicated to reviewing our manuscript. We are pleased to hear that the improvements made have enhanced the quality of the paper to their satisfaction. We also appreciate the kind wishes for the publication process.
